# A Lincomycin-Specific Antibody Was Developed Using Hapten Prediction, and an Immunoassay Was Established to Detect Lincomycin in Pork and Milk

**DOI:** 10.3390/foods13193118

**Published:** 2024-09-29

**Authors:** Yuhan Shang, Dandan Zhang, Yun Shen, Yuanhu Pan, Jing Wang, Yulian Wang

**Affiliations:** 1 National Reference Laboratory of Veterinary Drug Residues (HZAU), Huazhong Agricultural University, Wuhan 430070, China; syhyimen@163.com (Y.S.); danzh420@163.com (D.Z.); m13670964790@163.com (Y.S.); panyuanhu@mail.hzau.edu.cn (Y.P.); 2 National Nanfan Research Institute (Sanya), Chinese Academy of Agricultural Sciences, Sanya 572024, China; 3 Institute of Quality Standard and Testing Technology for Agro, Products, Chinese Academy of Agricultural Sciences, Key Laboratory of Agro-Product Quality and Safety, Ministry of Agriculture Beijing, Beijing 100081, China

**Keywords:** lincomycin, monoclonal antibody, ELISA, milk, pork

## Abstract

Prolonged consumption of animal-derived foods containing high levels of lincomycin (LIN) residues can adversely impact human health. Therefore, it is essential to develop specific antibodies and immunoassay methods for LIN. This study utilized computational chemistry to predict the efficacy of LIN haptens prior to chemical synthesis, with subsequent confirmation obtained through an immunization experiment. A hybridoma cell line named LIN/1B11 was established, which is specific to LIN. The optimized indirect competitive enzyme-linked immunosorbent assay (ic-ELISA) method exhibited high specificity for detecting LIN residues, with an IC50 value of 0.57 ± 0.03 µg/kg. The method effectively detected LIN residues in pork and milk samples, achieving a limit of detection (LOD) ranging from 0.81 to 1.20 µg/kg and a limit of quantification (LOQ) ranging from 2.09 to 2.29 µg/kg, with recovery rates between 81.9% and 108.8%. This study offers a valuable tool for identifying LIN residues in animal-derived food products. Furthermore, the efficient hapten prediction method presented herein improves antibody preparation efficiency and provides a simple method for researchers in screening haptens.

## 1. Introduction

The alkaline lincomide antibiotic, known as lincomycin (LIN), was first discovered in 1962 through isolation from *Streptomyces lincolnensis* [1]. Its composition primarily consists of amino acids, specifically, L-proline substituted with a 40-alkyl chain and a sugar molecule connected through an amide linkage [2]. In 1967, LIN was granted approval in the United States for treating Gram-positive bacterial infections, thus broadening its application to veterinary medicine for various infections [3]. According to the *Chinese Veterinary Pharmacopoeia (2020)*, LIN is primarily indicated for treating Gram-positive bacterial infections and is effective against porcine treponema and mycoplasma infections [4]. The primary mechanism of action of LIN involves targeting the 50S ribosomal subunit in susceptible bacteria [5]. LIN occupies a specific site in the region where the bacterial ribosome extends the nascent peptide chain [6]. The 3’ end of the simulated (de)acetyl-tRNA inhibits protein synthesis during the initial phase of the peptide chain extension cycle, thereby exerting antimicrobial activity [7]. As the main representative of lincosamides (LINS), LIN is ranked third in usage in China and plays an important role in veterinary medicine [8]. While LIN itself has relatively low toxicity, excessive consumption of animal-derived food containing the LIN residue can pose a risk to human health, potentially leading to pseudomembranous enteritis [9]. Additionally, it can contribute to the emergence of bacterial resistance to LIN [10].

Many countries have established maximum residue limits (MRLs) for LIN in animal-derived food products to safeguard human health. Within the European Union, MRLs for LIN were set at 100 µg/kg in muscle tissue, 50 µg/kg in fat tissue, 500 µg/kg in liver tissue, 1500 µg/kg in the kidney, and 150 µg/kg in milk, respectively [11]. According to the national food safety standard of China (GB 31650-2019) [12], MRL for LIN in cattle and sheep was set at 50 µg/kg, while for pigs, it was established at 100 µg/kg.

The predominant analytical methods utilized for the detection of LIN residues in animal-derived food products encompass high-performance liquid chromatography (HPLC) [13], gas chromatography–mass spectrometry (GC–MS) [14], liquid chromatography–tandem mass spectrometry (LC–MS/MS) [15,16], and ultra-high-performance liquid chromatography–tandem mass spectrometry (UHPLC–MS/MS) [17]. As shown in Table S1 [18,19,20,21,22,23,24,25,26,27,28,29,30,31], the detection limits of GC and GC–MS/MS are relatively high, requiring intricate processes. In contrast, LC–MS demonstrates exceptional sensitivity but necessitates the use of the internal standard method for quantification, resulting in higher costs. The application of instrumental analysis methods for the detection of veterinary drug residues is associated with certain limitations, including intricate processing procedures, costly instrumentation, and prolonged detection timeframes, all of which impede the timely on-site analysis of samples.

The immunoassay method compensates for the limitations of instrumental analysis, exhibiting characteristics such as simplicity, high efficiency, and cost-effectiveness. Consequently, it has gained widespread application in food safety detection and other fields, significantly reducing the pre-processing workload associated with large sample quantities while enabling rapid on-site sample detection [32]. In recent years, there has been a growing interest in utilizing immunoassay methods for the detection of LIN residues in animal-derived food, especially the enzyme-linked immunosorbent assay (ELISA) method, evidenced by the data presented in Table 1. Both domestic and international ELISA methods for LIN involve complex hapten synthesis, requiring substantial manpower and resources, which adds to the overall complexity of the method. Hapten design is a key step in antibody preparation and is crucial for antibody production [33]. At present, LIN hapten design strategies are primarily based on experience, which tend to be inefficient. Recently, computer-assisted modeling technology has become an effective tool for rational hapten design and the research of cross-reaction rates (CR) [34].

In this study, we selected three typical haptens, L1–L3, as models to predict their suitability for generating specific antibodies against LIN using computational chemistry. The antiserum titer and affinity among the immunization groups from the three haptens were compared to evaluate how the hapten structures affect the characteristics of the obtained antisera. Finally, based on the hapten L2, we synthesized a novel hapten and developed a mAb 1B11 with specificity for LIN. An indirect competitive enzyme-linked immunosorbent assay (ic-ELISA) method was established for the detection of LIN in pork and milk. This advancement provides a convenient and simplified approach to drug residue detection.

## 2. Materials and Methods

### 2.1. Chemicals

Lincomycin hydrochloride (LIN, 98%), N, N’-dicyclohexylcarbodiimide (DCC, 99%), N, N’-carbonyldiimidazole (CDI, 99%), 4-dimethylaminopy-ridine (DMAP, 98%), Ethyl 3-mercaptopropionate (96%), Triphenylchloro-methane (TPCM, 97%), Acrylic Acid (AA, 98%), and sodium periodate (NaIO_4_, 99.5%) were provided by Shanghai McLean Biochemical Technology Co., Ltd. (Shanghai, China). Clindamycin hydrochloride (CLIN, 98%) was purchased from Shanghai Haohong Biopharmaceutical Technology Co., Ltd. (Shanghai, China). Pirimicin (PIR, 96%) was acquired from the Beijing North Weiye Metrology Technology Research Institute (Beijing, China). N-hydroxysuccinimide (NHS) was purchased from China National Pharmaceutical Group Chemical Reagent Co., Ltd. (Beijing, China). Bovine serum albumin (BSA), Ovalbumin (OVA), Horseradish peroxidase (HRP)-labeled sheep anti-mouse IgG, Polyethylene glycol (PEG), Freund’s complete adjuvant (FCA), Freund’s incomplete adjuvant (FIA), HAT medium (50×), and HT culture medium (50×) were purchased from Sigma. All other chemical reagents were analytically pure and commercially chemically available. The milk and pork were purchased from the local supermarket of Huazhong Agricultural University.

### 2.2. Computational Chemistry Analysis of Haptens

To reveal the rationality of the hapten design, the lowest energy conformations of L1–L3 were built in the Chem 3D 14.0.0.17 software [42]. The atomic charge distribution of the haptens was directly extracted from the output file. Then, M06–2X density functional theory calculations combined with the TZVP basis set were executed to optimize the haptens using the Gaussian 09 package (Gaussian, Wallingford, CT, USA). GaussianView 5.0 was used to analyze the electrostatic potential (ESP) surfaces [43]. The alignments of the hapten and protodrug minimum energy constructs were investigated by molecular docking using SYBYL-X 2.0 software [44].

### 2.3. Cells and Animals

Female Balb/c mice, aged 6–8 weeks, were obtained from the Laboratory Animal Center of China Three Gorges University (registration number: NO.42010200005556) and used in accordance with the approved animal testing ethics protocol (HZAUMO-2022-0116).

The SP2/0 myeloma cells in mice were preserved in liquid nitrogen at the national veterinary drug residue reference laboratories at Huazhong Agricultural University.

### 2.4. Synthesis of Haptens

**Figure 1:**
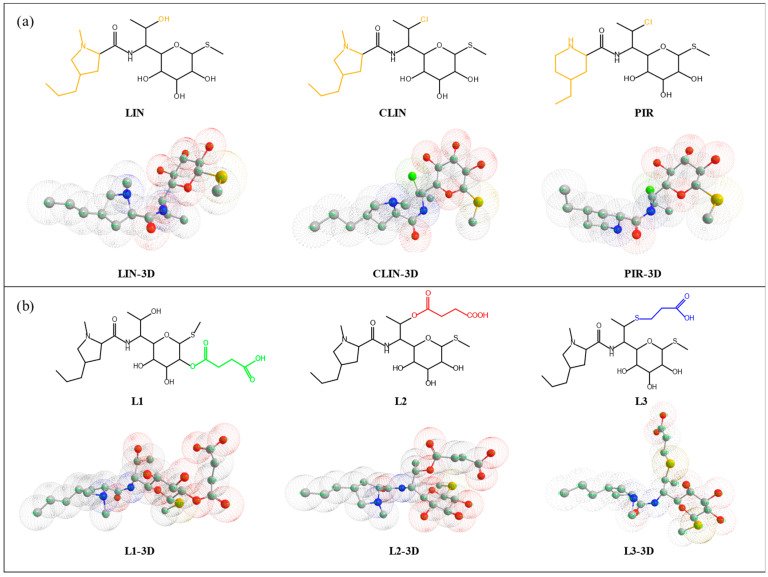
(**a**) The chemical structure and minimum energy profile of LINS. The lowest energy conformation of the drug: red, oxygen; blue, nitrogen; gray, carbon; yellow, sulfur; green, chlorine. (**b**) The chemical structures and minimum energy profiles of three haptens. The lowest energy conformation of the drug: red, oxygen; blue, nitrogen; gray, carbon; yellow, sulfur.

**Figure 2:**
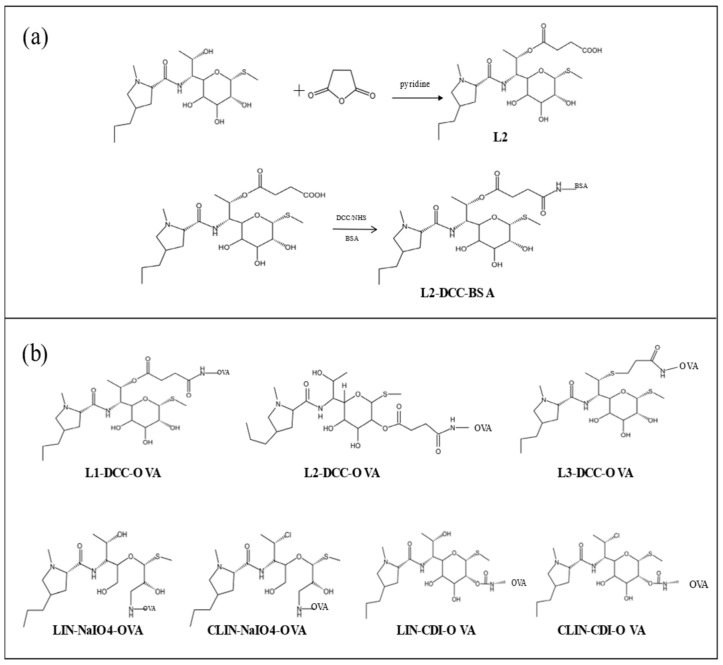
(**a**) The synthetic pathway of L2-DCC-BSA. (**b**) The structural formula of seven coating antigen chemicals.

LIN hydrochloride (6 g) underwent a reaction with saturated NH_4_Cl, resulting in the formation of an intermediate that was subsequently extracted using n-butanol and evaporated to yield L2 (Figure 1b). The synthesis steps of hapten L2 is shown (Figure 2a). The molecular weight of hapten L2 was determined to be 506.23. The structural formula of hapten L1 is shown (Figure 1b). The structural formula of hapten L3 is shown (Figure 1b).

### 2.5. Synthesis of Antigens

The immunogen L1-DCC-BSA was synthesized by coupling L1 (15.3 mg) with BSA using the DCC/NHS method [9]. Due to the presence of a carboxyl group at the C7 position in the L2 molecule, it was capable of being conjugated to the carrier protein BSA using the carbodiimide method (DCC/NHS). Fifty-eight milligrams of hapten, 10 mg of NHS, and 18 mg of DCC were weighed and dissolved in 1.8 mL of DMF. The mixture was stirred overnight at room temperature and subsequently filtered to yield the supernatant A solution. According to the method, we took 112 mg of BSA dissolved in 14 mL of PBS; this is for solution B. Under ice bath conditions, solution A was slowly added drop by drop to solution B, and the reaction was stirred overnight. The reaction solution was dialyzed in PBS at 4 °C for 3–5 days, with the dialysate changed three times daily before being transferred to centrifuge tubes. We centrifuged at 10,000 r/min for 10 min, discarded the precipitate and took the supernatant on standby. The immunogen L3-DCC-BSA was generated by coupling L3 (10.25 mg) with the BSA [42]. The resulting immunogens were dialyzed and stored at a temperature of −20 °C.

This present study involved the synthesis of seven different types of coating antigens (Figure 2b). The carboxylic groups present in L1, L2, and L3 enable their conjugation with the carrier protein OVA using the carbodiimide method (DCC/NHS).

The inclusion of hydroxyl groups in LIN and CLIN facilitates the synthesis of full antigens through the utilization of the CDI and NaIO_4_ methodologies. The activation solution was formulated by dissolving LIN and CDI in 1 mL of DMF, which was subsequently added dropwise to the carrier protein solution at an ambient temperature for a duration of 10 h. The initial LIN-CDI-OVA compound was successfully obtained. The procedure for synthesizing the coating antigen CLIN-CDI-OVA closely mirrors that of synthesizing the coating antigen LIN-CDI-OVA. Through the utilization of the sodium periodate (NaIO_4_) reduction method, the adjacent hydroxyl groups on LIN pyranose were effectively oxidized to aldehyde groups, which subsequently underwent a condensation reaction with the free amino group of OVA, resulting in the acquisition of the initial LIN-NaIO_4_-OVA product. The synthesis of the coating antigen CLIN-NaIO_4_-OVA closely follows the same protocol as the coating antigen LIN-NaIO_4_-OVA. A total of 7 different types of coating antigen were successfully synthesized.

### 2.6. The Detection Mode of ELISA

In this experiment, the indirect ELISA method was employed to ascertain the titer, while the indirect competitive ELISA method was utilized to evaluate the inhibition. The iELISA method was conducted by diluting the coating antigen with CBS to a working concentration of 100 µL per well and incubating it at 4 °C overnight. Following incubation, the coating antigen was discarded, and 250 µL of the washing solution was added to each well. After allowing the solution to stand for 30 s, it was removed, and the wells were patted dry. This washing procedure was repeated three times. A total of 250 µL of 1% blocking solution was added to each well, which was subsequently sealed in a humidified chamber at 37 °C for 2 h. After discarding the block buffer, the sample was washed three times and dried. The antibody was diluted in PBS to 100 µL per well and incubated for 40 min at 37 °C in a humidified box. The HRP-labeled sheep anti-mouse secondary antibody was diluted in PBS to a working concentration of 1:500. A volume of 100 µL per well was then incubated for 40 min at 37 °C in a humidified chamber. Substrate (100 µL/well) was added and incubated for 15 min. Then, 50 µL of the termination solution was added per well, and the absorbance at 450 nm (OD_450_) was measured using a microplate reader.

The ic-ELISA followed the same steps as the iELISA. Following the application of the blocking buffer, 50 µL of the serially diluted standard and 50 µL of the antibody were added to each well, allowing the standard drug to compete with the drug on the microplate for the antibody.

### 2.7. Animal Immunization

The immunization protocol for eight female BALB/c mice included primary immunization, booster immunization, and induction of abdominal immunity. The mice were administered immunogen doses of 50 μg and 100 μg, respectively. The immunization regimen included a primary immunization and multiple booster immunizations. The first primary immunization was emulsified using Freund’s complete adjuvant, and the mice were injected subcutaneously at multiple points on the back of the neck. After 21 days, the booster immunization was performed with Freund’s incomplete adjuvant, and the emulsified antigen was intraperitoneally injected into the mice. The interval between each booster immunization was 14 days. The serum titers were assessed via iELISA in order to identify the mice exhibiting the greatest potency and specificity for intraperitoneal immunization.

### 2.8. Preparation of Monoclonal Antibodies

We prepared SP2/0 myeloma cells and immune spleen cells, fused them using PEG, cloned the hybridoma cells by limited dilution, screened for positive hybrids, and selected a high-titer, well-formed single colony for further cloning [45]. Once the hybridoma-positive rate on the 96-well board reached 100%, the cell culture was expanded, and cryopreservation was initiated.

### 2.9. Standard Curve and Specificity Determination

A standard curve was created based on the best coating concentration and antibody dilution. LIN was formulated into a series of gradients of concentrations, with five parallel sets for each concentration. The logarithm of the LIN concentration was used as the abscissa, and the inhibition rate B/B0 was used as the ordinate. The standard curve was drawn to obtain the regression equation and the correlation coefficient, and the IC_50_ value was calculated according to the regression equation. Cross-reactivity (CR) was determined using Formula 1. The magnitude of the cross-reactivity value indicates the level of recognition specificity of antibodies towards the drug in the evaluation method.

CR% = IC_50_ (LIN)/IC_50_ (Other Medications) × 100% (1)

### 2.10. Sample Preparation

Pork samples were subjected to grinding, followed by extraction with a mixture of methanol and 1.5 mmol/L of HCl (*v*/*v* = 1:6). The resulting supernatant was used for analysis. An identical pre-treatment procedure was also employed for the milk samples [9].

### 2.11. Validation of the ic-ELISA

The coefficient of variation within batches and the variation coefficient between batches were examined to assess the precision of the standard curve in the experiment. Five intra-plate and five inter-plate replicates were performed. The measured OD_450_ nm values were placed into the standard curve, and the actual measured values, intra-plate and inter-plate variation coefficients, were calculated to evaluate the precision of the standard.

## 3. Results

### 3.1. Analysis and Prediction Hapten Structure by Computational Chemistry

The molecular simulations of the LIN and LINS haptens were carried out utilizing the ChemBioDraw Ultra 12 simulations employing MM2 energy minimization to generate the minimum energy maps [46]. The lowest energy configuration of the system is depicted in Figure 1. As shown in Figure 1a, the different structures of LINS were marked in orange. The LINS differ less in structure, with all having 3-4 hydroxyl groups. The LINS different hydroxyl groups bridging different connection arms will form different haptens. Because PIR is expensive, LIN and CLIN were used as raw materials to synthesize different haptens. L1 and L2 utilized LIN as the primary raw material for haptens synthesis. The hapten LI was synthesized by linking the long arm of the linear chain at position C2. Hapten L2 was synthesized by linking the long arm of the linear chain at position C8. CLIN was the raw material of L3 as to the synthesis of hapten. In order to synthesiz L3, the long arm of the linear chain was connected at position C8. The connecting arms of the three haptens were marked in different colors. The minor structural changes in the hapten may significantly affect the antibody response [16]. Therefore, selecting proper hapten for the production of high-specificity LIN antibodies was of crucial importance [47].

**Figure 3:**
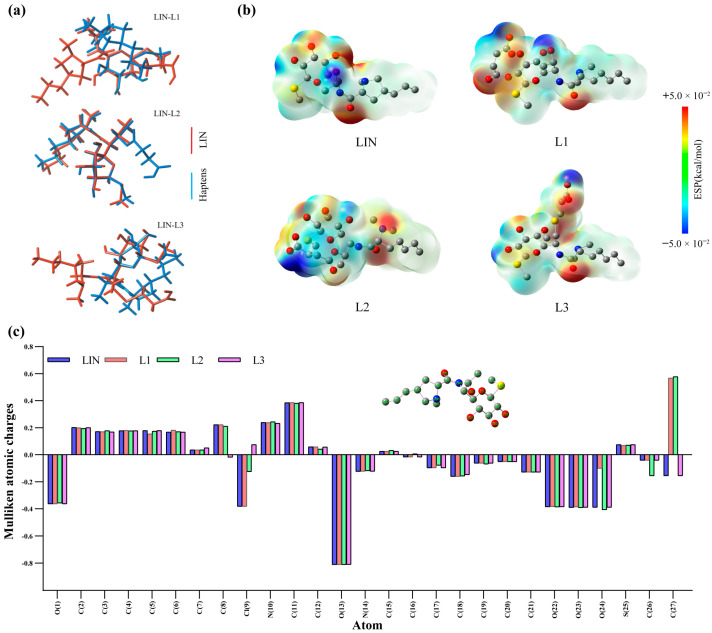
(**a**) The diagram illustrates the overlapping structure of LIN and haptens in three dimensions. (**b**) ESPs on the van der Waals surface of LIN and three haptens. The negative ESP regions were indicated in blue, the positive regions in red, and the neutral regions in green. (**c**) Atomic charge distribution of LIN and three haptens.

The objective of this present study was to generate specific mAbs that recognize LIN. As shown in Figure 3a, a three-dimensional comparative analysis was performed, and the minimum energy schematics of LINS and three haptens were superimposed, respectively. This analysis revealed that LIN and L2 exhibited the highest overlap rate [48]. The L1 and L3 in C2 and C8 connected different long arms, and the spatial structures changed. The space structures of L1 and L3 changed, resulting in a low overlap with LIN. Because the recognition mechanism of the antigen–antibody interactions was primarily attributed to electrostatic interactions, the Mulliken atomic charges of L1–L3 were calculated. As shown in Figure 3c, by comparing the charges of LIN and the haptens, the changes in antigenic determinants and atomic charges were obtained. The charge distribution of the main atoms was calculated based on the diagram depicting the lowest energy configuration. Significant discrepancies were observed in the charge disparities of L1 and L2, derived from LIN, at positions O9, O24, and C26. Significant discrepancies were observed in the charge disparities of L3 at positions C7 and C9. The ESPs in Figure 2b verified that the electronic distribution of the immunized haptens was different [49]. The more strongly negative regions of the three haptens were different. Changes in the negatively electric regions of L1 and L3 rotate the amide bond, which disrupts the recognition of the LIN backbone by the immune system. Therefore, the computational chemistry findings support that hapten L2 may be more conducive to eliciting a specific antibody response for LIN. The rationality of the hapten prediction was then further evaluated by chemical synthesis and animal experiments.

### 3.2. Characterization of Haptens

The L1, L2, and L3 were analyzed using time-of-flight–mass spectrometry (TOF–MS) with tandem mass spectrometry (MS/MS) (TOF–MS/MS) in the positive ion H+ mode. The hapten L1 exhibited a relative molecular weight of 506.23, with a [M+H]+ peak detected at *m/z* 507.25 (Figure S1). Similarly, the semi-antigen L2 had a relative molecular weight of 506.23, with a [M+H]+ peak observed at *m/z* 507.27 (Figure S2). The L3, with a relative molecular weight of 494.21, was analyzed using mass spectrometry in the positive ion H+ mode, revealing a detected [M+H]+ peak at *m/z* 495.21 (Figure S3). The results showed different fragmentation patterns for haptens L1 and L2, suggesting structural differences between the two compounds. The successful synthesis of L1, L2, and L3 has been accomplished.

### 3.3. Characterization of Conjugates

Complete conjugates were identified using SDS-PAGE. The results showed that compared with the BSA, the three immunogens all lagged behind, indicating that the immunogen synthesis was successful (Figure S4). The bands observed in the figure correspond to the biomarkers OVA, LIN-CDI-OVA, LIN-NaIO4-OVA, CLIN-NaIO4-OVA, L1-DCC-OVA, L2-DCC-OVA, L3-DCC-OVA, OVA, and CLIN-CDI-OVA, from left to right (Figure S5). The identification process of the seven original coating OVA bands during the SDS-PAGE analysis was slightly delayed due to the higher relative molecular weight of the original coating compared to that of the OVA. The band L1-DCC-OVA displayed a relatively light color and low protein concentration, which was attributed to the precipitation phenomenon resulting from the coupling process between L1 and OVA. A band lag relative to OVA was consistently observed in all seven coatings, indicating the successful synthesis of these coatings.

### 3.4. Characterization of the Antisera and the Monoclonal Antibody

**Figure 4:**
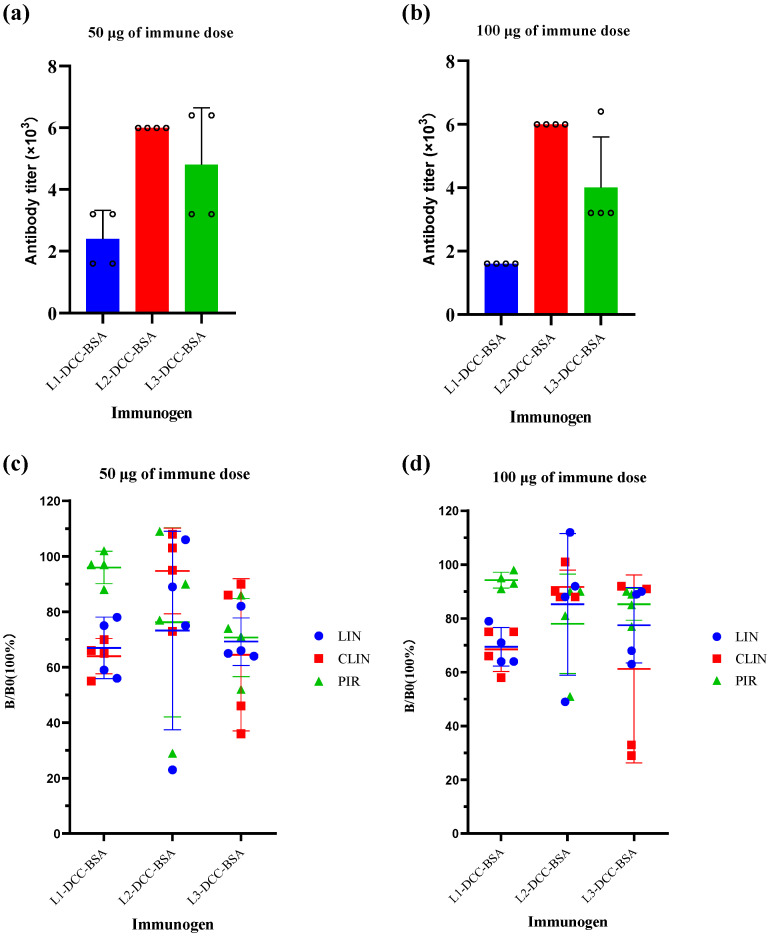
(**a**) Serum titer of four mice with an immune dose of 50 μg. (**b**) Serum titer of four mice with an immune dose of 100 μg. (**c**) Sensitivity of mouse serum with an immune dose of 50 μg. (**d**) Sensitivity of mouse serum with an immune dose of 100 μg.

The immune response of mice directly impacts the efficacy of monoclonal antibodies. The immunogens L1-DCC-BSA, L2-DCC-BSA, and L3-DCC-BSA were administered at doses of 50 μg and 100 μg, respectively. Following immunization, the serum titers were detected. The results indicate that the serum titer of immunogen L2-DCC-BSA displayed a superior immune effect (Figure 4a,b). Concurrently, the serum inhibition rates were evaluated, revealing that the serum inhibition rate of immunogen L2-DCC-BSA was the lowest among all samples examined (Figure 4c,d). Thus, the research findings indicated that hapten L2 works best and is consistent with the prediction results of hapten—a correlation between the immune response of mice and the dosage of immunization [50]. The immune response of the mouse body may be compromised if the dosage of immunization is too low, while an excessive dosage may lead to immunosuppressive effects. The antibody titer generally increases within a specific range as the immune dose increases.

The chromosome count of the 1B11 hybridoma cell line should align with the total number of chromosomes in spleen cells and SP2/0 cells. The karyotype analysis of the 1B11 hybridoma cell line is illustrated in Figure S6. Ten 1B11 hybridoma cell lines were analyzed, demonstrating an average chromosomal count of 102.6, as shown in Table S2, confirming the successful fusion and formation of a hybridoma cell line.

The monoclonal antibody was produced using the in vivo induced ascites tumor method and was characterized using UV spectroscopy. The protein exhibited a maximum absorption peak at 279 nm (Figure S7). The concentration of antibodies measured was 32.24 mg/mL (Figure S8). The 1B11 antibody subtype was classified as IgG1 (Figure S9).

### 3.5. Optimization and Standard Curve for the ic-ELISA

The coating antigen has been demonstrated to play a pivotal role in enhancing detection sensitivity. The coating antigen’s structure directly impacts the immune methods’ detection performance [19]. The results of multiple studies have shown that a significant difference in the structural composition of the synthesized coating antigen compared to the immunogen can improve the sensitivity of immune assays [51,52].

This study aimed to improve detection sensitivity by evaluating seven synthesized coating antigens and ultimately selecting LIN-NaIO4-OVA (Figure 3a–c) as the optimal choice due to its unique hexagonal sugar structure and open-loop configuration, which differed significantly from the immunogen’s structure and showed exceptional sensitivity. By optimizing coating antigens, the coating antigens that are structurally different from immunogen were found to be crucial in enhancing the sensitivity of ic-ELISA.

**Figure 5:**
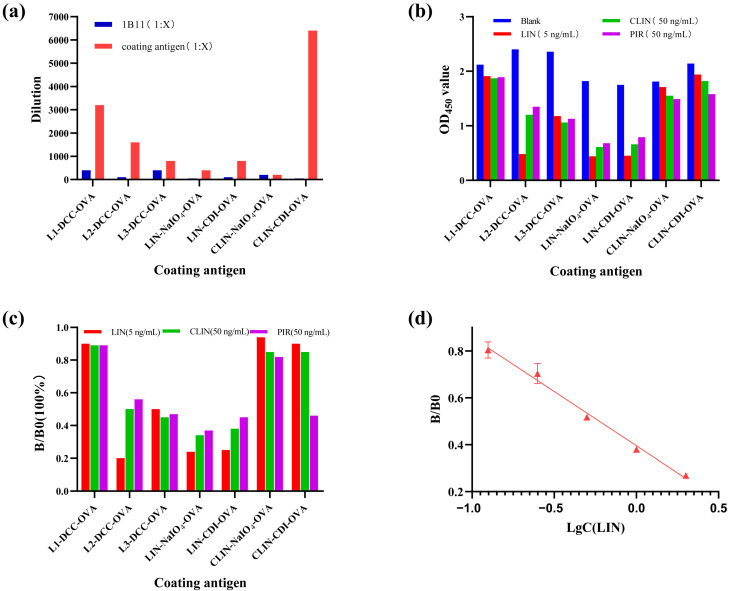
(**a**) The optimal concentrations of seven coating antigens and the optimal dilution of antibody 1B11. (**b**) Comparison of OD_450_ value of seven coating antigens. (**c**) Comparison of B/B_0_ value of seven coating antigens. (**d**) Standard curve of ic-ELISA for LIN.

The ELISA standard curve for LIN was generated using the ELISA Calc software for linear regression analysis. As depicted in Figure 5d, LIN standards were prepared. The results showed a regression equation of Y = 0.402 − 0.406X and an R^2^ value of 0.9953. The IC50 value was calculated to be 0.57 ± 0.03 µg/kg within the linear range of 0.125–4 µg/kg. The calculated sensitivity was 0.091 ng/mL. The results are shown in Table S3. Table S4 demonstrates that the method exhibits commendable precision, with an intra-plate coefficient of variation (CV) of ≤19.0% and an inter-plate CV of ≤16.4%. Compared with the ELISA established by Wang et al., the IC50 was increased by 2–60 times.

This study evaluated the cross-reactivity of the 1B11 monoclonal antibody to LINS compounds. A standard curve was successfully established using optimized conditions for ELISA. The cross-reactivity rates of the 1B11 monoclonal antibody were found to be 2.8% for CLIN and 2.4% for PIR (Table S5).

### 3.6. Elimination of Matrix Effect

The pork was minced, 2 g were placed in a centrifuge tube, mixed with a LIN standard solution, vortexed for 5 min, and then 10 mL of methanol/0.0015 mol HCl (1:6 *v*/*v*) was added and vortexed again for 5 min. We took 1 mL of milk and sequentially added the LIN standard solution to the milk sample. Then, we vortexed the mixture for 5 min. Subsequently, we added 9 mL of a methanol/0.0015 mol HCl solution (*v*/*v* = 1:6) and vortexed the mixture for 5 min. Finally, we centrifuged the plates at 5000 rpm for 5 min. The supernatant was taken, processed and detected by the method established in this study. To eliminate the matrix effect of pork and milk, the pork samples were diluted six times with the extract and the milk extract was diluted ten times with the sample extract. The findings are presented in Table 2.

### 3.7. Sample Preparation and Validation of the ic-ELISA Method

A series of tests were conducted on 20 samples of pork and milk sourced from various supermarkets. The LIN standard solution was added to the pork and milk samples according to the quantitative limits of 1 × LOQ, 2 × LOQ, and 4 × LOQ. Then, the samples were processed, and the supernatant was collected for detection by the method established in this study. The first aim was to determine the actual concentration of the blank samples of pork and milk and then calculate the LOD and LOQ for each sample. The LOD and LOQ values of LIN in pork were 1.20 µg/kg and 2.29 µg/kg, respectively. The LOD and LOQ values of LIN in milk were identified as 0.81 µg/kg and 2.09 µg/kg, respectively (Table 3). The recovery rates were between 81.9% and 108.8% (Tables S6 and S7).

### 3.8. Comparison with Other Immunoassays

The optimal hapten was determined through the utilization of ChemBioDraw Ultra 12, SYBYL-X 2.0, and GaussianView 5.0 software. The enzyme-linked immunosorbent assay (ELISA) method developed in this study demonstrates high sensitivity for the detection of LIN, with an IC_50_ value of 0.5 ng/mL. In comparison, the sensitivity of the ELISA method for the LIN detection reported by He et al. (2017) was 1.3 ng/mL [36], while that reported by Burkin et al. (2010) was 9.15 ng/mL [35]. Additionally, the GICA method for the LIN detection described by Guo et al. (2019) exhibited a sensitivity of 3.27 ng/mL [41].

## 4. Conclusions

In this study, a hapten prediction strategy for LIN was proposed to produce mAbs with high specificity. Computational chemical analyses of the lowest energy conformation and electrostatic properties were conducted to predict the rationality of three hapten models. The results revealed that hapten L2 introduced a spacer arm in the C8 position of LIN, and LIN exhibited the highest degree of overlap. Hapten L2 was proven to be favorable for the generation of high-quality antibodies to LIN, both by computational chemistry and antiserum studies—aligning with the observation of a high-specificity LIN monoclonal antibody generated through L2. In contrast to the prior LIN haptens, the L2 synthesized in this study offers the benefits of straightforward synthesis and convenient operation. This also suggests that a greater degree of overlap between the hapten and drug corresponds to a higher specificity of the resulting antibody. Then, through a comparative analysis of the chemical structure formulas of seven coating antigens, it was determined that the hapten linked to LIN-NaIO4-OVA exhibited the most significant structural divergence from LIN. The results indicate that LIN-NaIO4-OVA was the most effective choice for coating antigens. This finding supported the notion that hetero-coated coating antigens could enhance detection sensitivity. In addition, a rapid, reliable ic-ELISA was established for the detection of LIN in real samples with satisfactory sensitivity and specificity.

## Figures and Tables

**Table 1:** Immunological assays for the detection of LIN.

Detection Method	Hapten	Target Analytes	IC_50_ (µg/kg)	LOD (µg/kg)	Source
ELISA	LIN derivative	LIN, CLIN	LIN: 29.1 CLIN:153.8	0.15–0.98	(Wang et al. 2010) [9]
ELISA	LIN	LIN, CLIN	LIN: 9.15 CLIN: 18.3	0.43–0.65	(Burkin et al. 2010) [35]
ELISA	CLIN-HS	LIN, CLIN	CLIN: 0.3 LIN: 1.2	1.8–6.8	(He et al. 2017) [36]
ELISA	PIR	PIR	1.6	1.65–4.45	(Jiang et al. 2016) [37]
FICA	LIN	LIN	2.2	0.69	(Zhou et al. 2014) [38]
ICA	CLIN-HS	CLIN	2.4	1.0	(Wang et al. 2016) [39]
GICA	LIN-HS	LIN, CLIN	LIN: 0.3 CLIN: 50	2.1	(Cao et al. 2015) [40]
GICA	CLIN chlorine substituents	LIN, CLIN, PIR	CLIN: 0.42 LIN: 3.27 PIR: 12.8	0.04–20.91	(Guo et al. 2019) [41]

**Table 2:** Recovery of LIN from artificially contaminated food samples.

		Recovery (%) for Samples Spiked at Concentrations		
Matrix	Sample Dilution	2.5	5	10
Pork	1:6	99.8 ± 7.8 ^a^	106.5 ± 2.3	102.5 ± 2.8
milk	1:10	94.9 ± 3.8	92.6 ± 10.6	92.6 ± 10.6

^a^ Standard deviation samples of foodstuffs (n = 3).

**Table 3:** LODs, LOQs, CV, and recovery of multiple LINs in real samples.

Targets	Samples	LOD	LOQ	Spiked Level (µg/kg)	Recovery (%)	CV (%)
		(µg/kg)	(µg/kg)			
LIN	pork	1.2	2.29	2.5, 5, 10	92.0–108.8	2.2–7.9
	milk	0.81	2.09	2.5, 5, 10	81.9–103.2	3.4–11.4
CLIN	pork	30.37	52.87	50, 100, 200	80.4–97.0	3.9–6.2
	milk	33.74	75.63	75, 150, 300	81.9–112.0	0.8–5.8
PIR	pork	28.03	55.92	60, 120, 240	69.7–100.0	11.3–16.6
	milk	52.96	106.62	100, 200, 400	72.2–104.0	11.0–14.2

## Data Availability

The original contributions presented in the study are included in the article and Supplementary Materials, further inquiries can be directed to the corresponding authors.

## Supplementary Materials

The following supporting information can be downloaded at: https://www.mdpi.com/article/10.3390/foods13193118/s1. Table S1 Research status of LIN; Table S2. Chromosome numbers of hybridoma cell lines; Table S3. Sensitivity of the ic-ELISA; Table S4. Coefficient of variability of intra-assay and inter-assay; Table S5. Cross-reactivity of 1B11 monoclonal antibody to LINS compounds; Table S6. Recoveries of LINS in pork; Table S7. Recoveries of LINS in milk; Table S8 Ic-ELISA date of standard curve for LIN; Figure S1. TOF–MS/MS spectrum of L1; Figure S2. TOF–MS/MS spectrum of L2; Figure S3. TOF–MS/MS spectrum of L3; Figure S4. Immunogen SDS-PAGE electrophoresis map; Figure S5. Coating original SDS-PAGE electrophoresis map; Figure S6. The chromosome of hybridoma cell (1000×); Figure S7. UV spectra of monoclonal antibodies; Figure S8. BCA kit for determining monoclonal antibody protein concentration; Figure S9. Subtype identification of 1B11.
