# Supplementary material for: A Lincomycin-Specific Antibody Was Developed Using Hapten Prediction, and an Immunoassay Was Established to Detect Lincomycin in Pork and Milk
Source: Foods. 2024 Sep 29;13(19):3118. doi: 10.3390/foods13193118 (PMC11476017; doi:10.3390/foods13193118)
Supplement: Supplementary file 1 [file foods-13-03118-s001.zip › foods-3146871-supplementary.pdf]

A Lincomycin-Specific AntibodyWas Developed Using Hapten Prediction, and an Immunoassay Was Established to Detect Lincomycin in Pork and Milk

Yuhan Shang [1,2], Dandan Zhang [1], Yun Shen [1], Yuanhu Pan [1], Jing Wang [2,3,*] and Yulian Wang [1,*]

[1] National Reference Laboratory of Veterinary Drug Residues (HZAU), Huazhong Agricultural University, Wuhan 430070, China

[2] National Nanfan Research Institute (Sanya), Chinese Academy of Agricultural Sciences, Sanya 572024, China

[3] Institute of Quality Standard and Testing Technology for Agro, Products, Chinese Academy of Agricultural Sciences, Key Laboratory of Agro-Product Quality and Safety, Ministry of Agriculture Beijing, Beijing 100081, China

* Correspondence: wangjing05@caas.cn (J.W.);

wangyulian@mail.hzau.edu.cn (Y.W.);

Tel.: +86-27-87287140 (Y.W.); Fax: +86-27-87672232 (Y.W.)

Table S1. Research status of LIN

Drugs	Method of detection	Samples	LOD (μg/L)	Recovery (%)	References
LIN	HPLC–MS/MS	Milk	1.0	101.0	(Chiesa et al 2016) [1]
LIN<br>CLIN	HPLC–MS/MS	Milk	LIN:1.0<br>CLIN:1.0	LIN:95.4-113.0<br>CLIN:90.6-105.0	(Zhao et al 2017) [2]
LIN	LC–MS/MS	Feed	4.0	80.0-111.8	(Jang et al 2022) [3]
LIN	HPLC	Feed	50.0	-	(Valese et al 2017) [4]
LIN	LC–MS/MS	Chicken	5.0	71.0-98.0	(Chiaochan et al 2010) [5]
LIN	LC–MS/MS	Duck	2.0	91.5-102.4	(Zheng et al 2019) [6]
LIN	HPLC–MS/MS	Honey	1.0	100.5-105.2	(Xiaoming et al 2021) [7]
LIN<br>CLIN	LC–MS/MS	Milk	LIN:18.7<br>CLIN:18.7	60.0-68.0	(Jank et al 2015) [8]
LIN	LC–MS/MS	Honey	-	69.0-75.0	(Lopez et al 2008) [9]
LIN	LC–MS/MS	Beef, Pork	3.0	71.0-102.0	(Carretero et al 2008) [10]
LIN	LC–MS/MS	Egg	1.6	99.4	(Spisso et al 2010) [11]
LIN<br>CLIN	LC–MS/MS	Chicken	3.0<br>3.0	94.0-104.0<br>104.0-115.0	(Bousova et al 2013) [12]
LIN	UPLC–MS /MS	Milk	1.0	85.6-106.0	(Wang et al 2016) [13]
LIN<br>CLIN	LC–MS/MS	Feed	LIN:.3.0<br>CLIN:2.0	56.1-98.3	(Suo et al 2018) [14]
LIN<br>CLIN	LC–MS/MS	Pork	-	41.4-52.4	(Xie et al 2012) [15]
LIN<br>CLIN<br>PIR	LC–MS/MS	Honey	-	93.0-98.0	(Bohm et al 2012) [16]
LIN<br>CLIN<br>PIR	HPLC–MS/MS	Pork, Beef	-	106.0-107.0	(Bohm et al 2011) [17]

Table S2. Chromosome numbers of hybridoma cell lines

Cell Line	Chromosome Number（n=10）										Average
1B11	103	108	108	106	102	109	106	102	104	108	102.6

Table S3. Sensitivity of the ic-ELISA

LIN (ng/mL)	OD_{450} of the standard curve					The measured value of the blank solution (ng/mL)				Average value (ng/mL)	SD (ng/mL)	LOD (ng/mL)
	1	2	3	4	5							
0	2.152	2.262	2.247	2.228	2.156	0.04	0.04	0.07	0.06			
4	0.393	0.442	0.356	0.378	0.412	0.07	0.07	0.05	0.09			
2	0.548	0.624	0.641	0.611	0.567	0.07	0.06	0.04	0.07	0.058	0.011	0.091
1	0.765	0.813	0.843	0.854	0.886	0.06	0.06	0.04	0.06			
0.5	1.12	1.215	1.241	1.132	1.231	0.04	0.04	0.07	0.06			
0.25	1.283	1.555	1.478	1.323	1.432							

Table S4. Coefficient of variability of intra-assay and inter-assay

LIN (ng/mL)	Measured values (ng/mL)	intra-CV (%)	Average value (ng/mL)	inter-CV (%)
0.125	0.11±0.19	7.6	0.14 ± 0.02	16.4
	0.14±0.01	7.6		
	0.17±0.02	8.9		
	0.16±0.04	19.0		
	0.12±0.01	6.5		
0.25	0.38±0.04	9.3	0.33±0.04	13.0

	0.28±0.03	12.5		
	0.28±0.03	11.0		
	0.28±0.04	8.1		
	0.33±0.01	4.4		
0.5	0.60±0.08	3.9	0.59±0.02	3.5
	0.62±0.13	5.6		
	0.59±0.12	8.3		
	0.56±0.13	7.5		
	0.59±0.11	2.8		
1	1.09±0.08	3.9	1.04±0.04	3.5
	1.04±0.13	5.6		
	1.01±0.12	8.3		
	1.00±0.13	7.5		
	1.08±0.11	2.8		
2	2.07±0.19	9.3	2.01±0.07	3.7
	1.97±0.21	10.7		
	2.01±0.22	11.2		
	1.90±0.17	9.2		
	2.12±0.09	9.1		

Table S5. Cross-reactivity of 1B11 monoclonal antibody to LINS compounds

Drug	IC_{50} (μg/kg)	CR（%）	Standard Curve	R^2	concentration range (μg/kg)
LIN	0.57	100.0	y = -54.756x+79.143	0.9956	0.125~4
CLIN	20.35	2.8	y = -0.486x + 1.226	0.9936	3.125~50
PIR	23.61	2.4	y = -0.362x+0.997	0.9984	3.125~50

Table S6. Recoveries of LINS in pork

Targets	Spiked level (μg/kg)	Recovery (%) (C±SD)	Within each batch (CV%, n=5)	Average (C±SD)	Between each batch (CV%, n=15)
LIN	2.5	108.3±4.2	3.9	99.8±7.8	7.9
		89.4±10.5	11.7		
		101.8±7.1	6.9		
	5	108.9±2.4	2.1	106.5±2.3	2.2
		107.2±6.3	5.8		
		103.3±6.9	6.7		
	10	106.2±5.2	4.9	102.5±2.8	2.7
		99.4±5.7	5.7		
		101.9±9.9	9.7		
CLIN	50	91.1±16.2	17.7	92.3±3.6	3.9
		88.6±6.3	7.1		
		97.1±7.8	8.0		
	100	97.0±17.1	17.7	91.7±5.3	5.8
		93.5±10.9	11.6		
		84.5±6.4	7.6		
	200	93.2±7.5	6.1	85.7±5.3	6.2
		81.8±12.0	14.6		
		81.9±12.1	14.8		
PIR	60	93.2±9.2	9.9	89.9±10.1	11.3
		90.9±9.79	10.8		
		85.4±11.3	13.2		
	120	79.6±15.6	19.6	83.1±13.4	16.6
		80.9±11.6	14.4		
		88.9±14.0	15.7		
	240	84.1±6.8	8.1	85.0±9.6	11.3
		87.2±8.7	10.0		
		83.8±13.3	15.8		

Table S7. Recoveries of LINS in milk

Targets	Spiked level (ng/mL)	Recovery (C±SD)	Within each batch (CV%, n=5)	Average recovery (C±SD)	Between each batch (CV%, n=15)

LIN	2.5	90.6±11.3	12.5	94.9±3.8	4.0
		94.2±9.0	9.6		
		99.8±11.7	11.7		
	5	100.5±11.	12.0	92.6±10.6	11.4
		99.6±6.3	6.9		
		77.7±16.7	17.6		
	10	81.1±4.5	5.6	84.8±2.9	3.4
		85.0±5.3	6.3		
		88.2±7.0	8.0		
CLIN	75	82.4±8.0	9.7	82.6±0.7	0.8
		83.4±3.8	4.5		
		81.9±7.3	8.9		
	150	86.6±10.0	3.5	90.1±3.2	3.5
		94.2±15.7	11.6		
		92.0±11.2	12.2		
	300	114.5±4.1	3.6	105.8±6.2	5.8
		100.6±6.5	6.5		
		102.3±5.2	5.1		
PIR	100	86.1±10.0	17.7	83.4±9.2	11.0
		87.4±9.6	11.0		
		76.6±8.1	10.5		
	200	87.2±9.0	10.4	92.3±11.0	11.8
		91.0±16.8	18.5		
		98.7±6.9	7.0		
	400	92.4±12.4	13.4	91.0±13.0	14.2
		88.7±13.1	14.7		
		91.8±13.3	14.5		

Table S8 Ic-ELISA date of standard curve for LIN

Batche	LIN (μg/L)					
s	0	0.125	0.25	0.5	1	2
1	2.203	1.827	S51.447	1.115	0.837	0.590

	2.014	1.630	1.616	1.047	0.775	0.544
	2.128	1.632	1.426	1.054	0.699	0.522
	2.078	1.589	1.340	1.111	0.812	0.556
	2.102	1.603	1.414	1.096	0.868	0.612
2	2.132	1.574	1.247	1.092	0.809	0.576
	2.219	1.838	1.482	1.144	0.844	0.599
	2.316	1.842	1.595	1.196	0.878	0.625
	2.254	1.590	1.323	1.172	0.855	0.608
	2.157	1.615	1.387	1.122	0.820	0.582
3	1.982	1.555	1.305	1.029	0.758	0.535
	1.907	1.695	1.350	0.991	0.724	0.515
	1.948	1.597	1.512	1.01	0.740	0.526
	2.095	1.719	1.510	1.089	0.796	0.566
	1.804	1.712	1.414	0.902	0.685	0.487
4	2.031	1.623	1.378	1.056	0.772	0.569
	1.996	1.695	1.582	0.998	0.758	0.579
	1.986	1.737	1.412	1.033	0.755	0.536
	2.036	1.663	1.545	1.059	0.773	0.550
	1.998	1.597	1.483	1.039	0.759	0.539
5	2.213	1.752	1.577	1.15	0.841	0.575
	1.958	1.600	1.545	1.018	0.744	0.509
	2.036	1.615	1.578	1.058	0.774	0.529

	2.078	1.597	1.414	1.081	0.780	0.551
	2.156	1.695	1.483	1.121	0.819	0.571

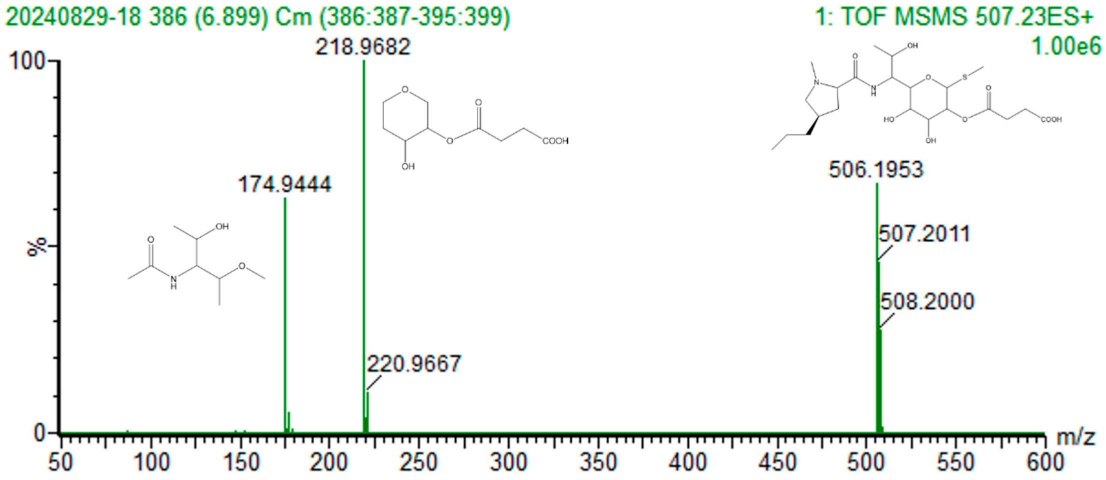

Figure S1. TOF–MS/MS spectrum of L1

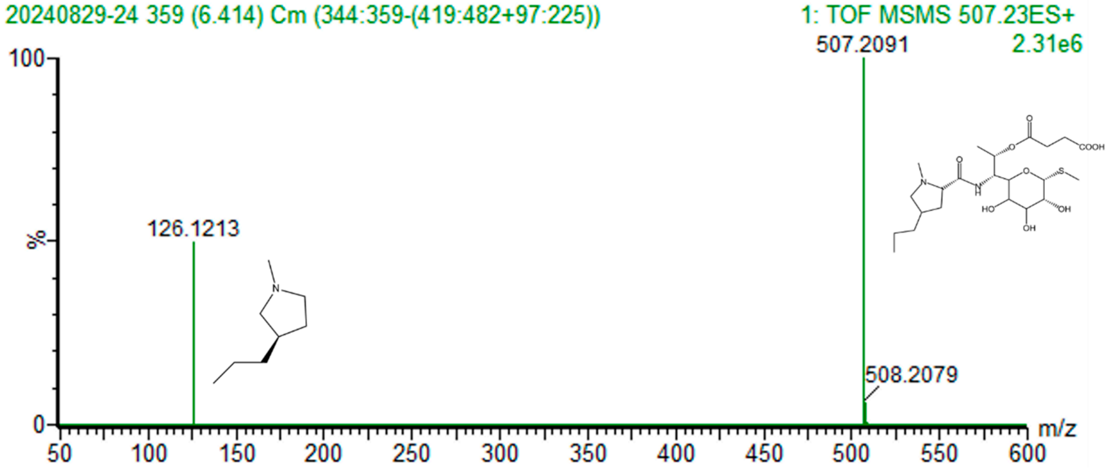

Figure S2. TOF–MS/MS spectrum of L2

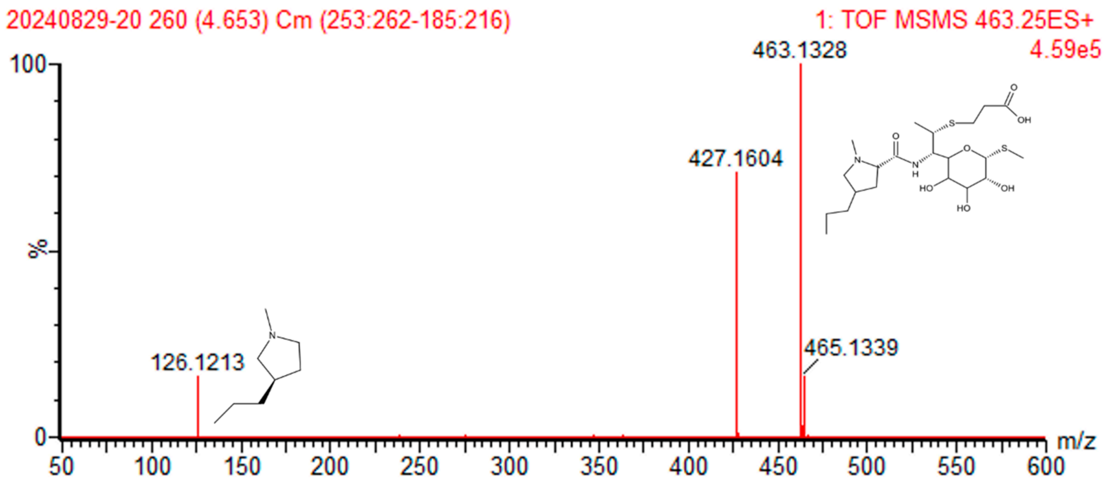

Figure S3. TOF–MS/MS spectrum of L3

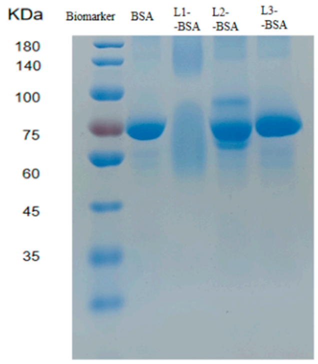

Figure S4. Immunogen SDS-PAGE electrophoresis map

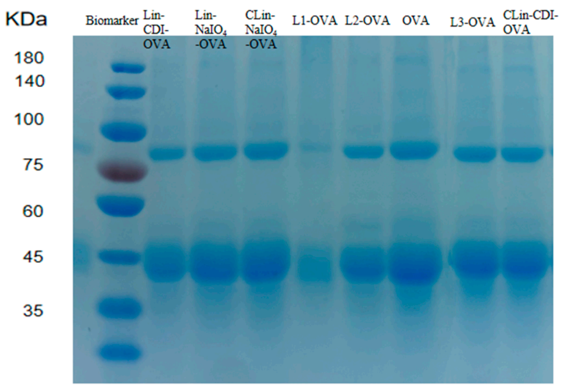

Figure S5. Coating original SDS-PAGE electrophoresis map

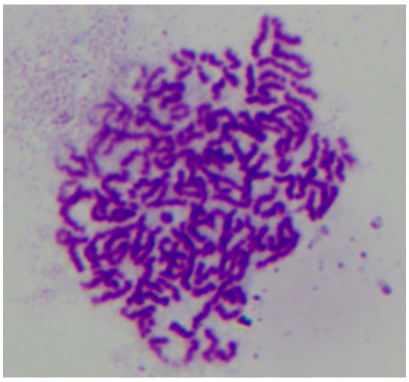

Figure S6. The chromosome of hybridoma cell（1000×）

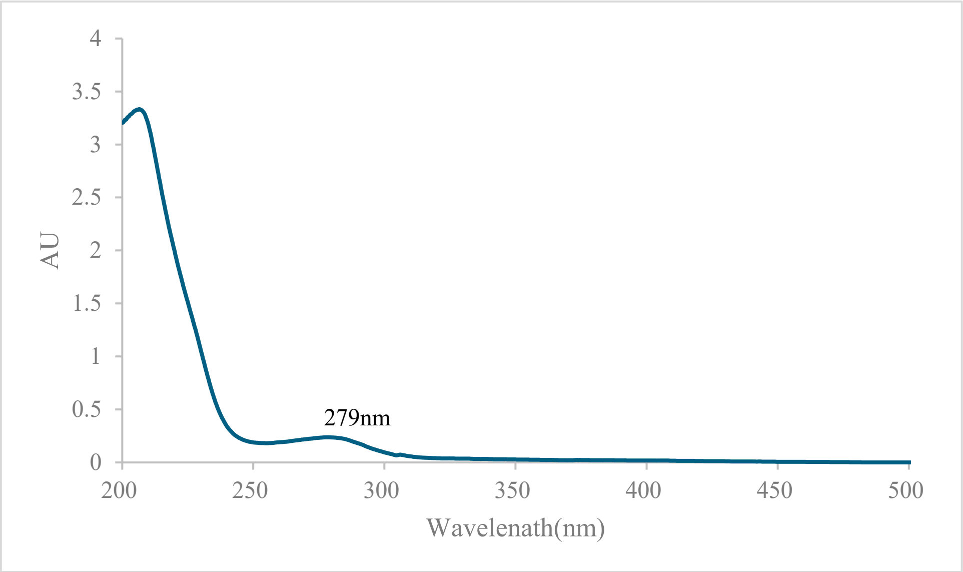

Figure S7. UV spectra of monoclonal antibodies

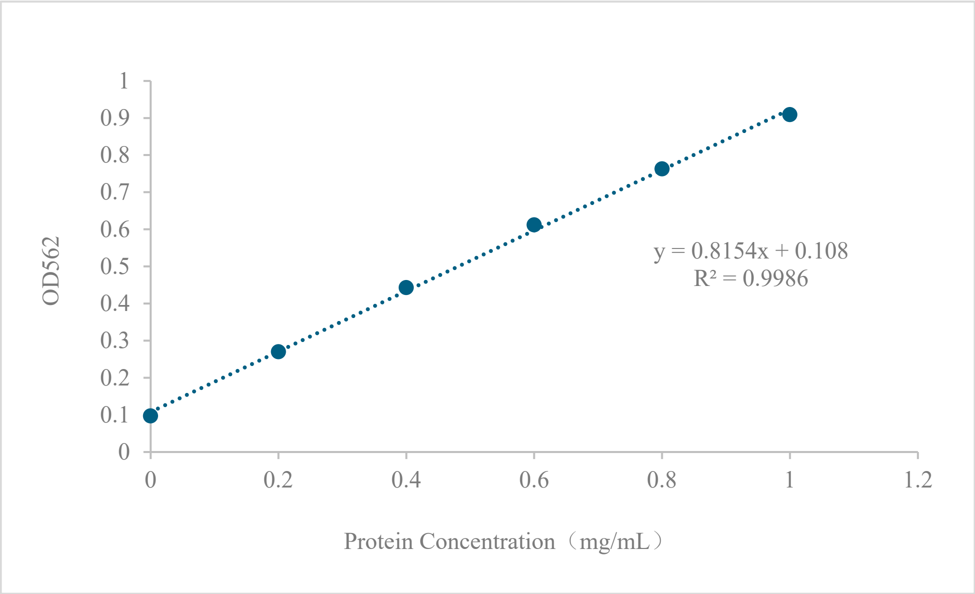

Figure S8. BCA kit for determining monoclonal antibody protein concentration

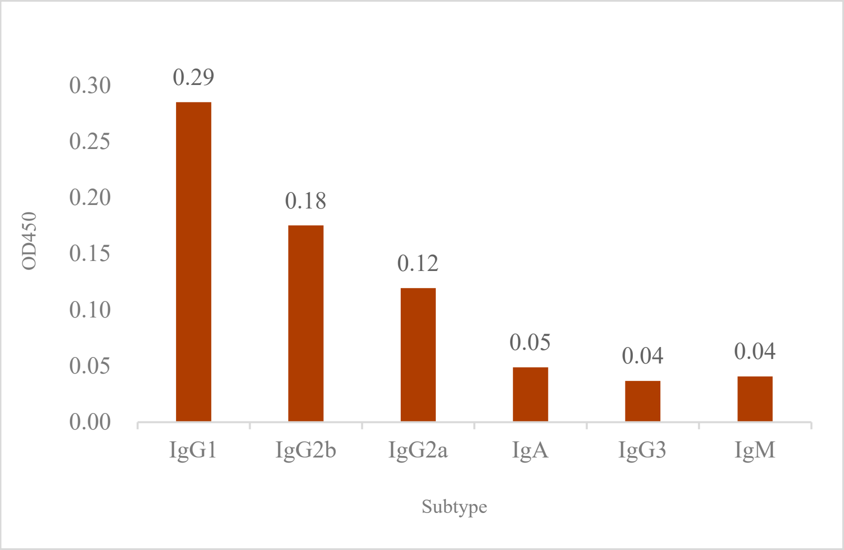

Figure S9. Subtype identification of 1B11
